# Resource Utilization Reduction for Evaluation of Chest Pain in Pediatrics Using a Novel Standardized Clinical Assessment and Management Plan (SCAMP)

**DOI:** 10.1161/JAHA.111.000349

**Published:** 2012-04-24

**Authors:** George R. Verghese, Kevin G. Friedman, Rahul H. Rathod, Amir Meiri, Susan F. Saleeb, Dionne A. Graham, Robert L. Geggel, David R. Fulton

**Affiliations:** From the Department of Cardiology, Children's Hospital Boston and the Department of Pediatrics, Harvard Medical SchoolBoston, MA

**Keywords:** chest pain, pediatrics, quality improvement, resource utilization

## Abstract

**Background:**

Chest pain is a common reason for referral to pediatric cardiologists. Although pediatric chest pain is rarely attributable to serious cardiac pathology, extensive and costly evaluation is often performed. We have implemented a standardized approach to pediatric chest pain in our pediatric cardiology clinics as part of a broader quality improvement initiative termed Standardized Clinical Assessment and Management Plans (SCAMPs). In this study, we evaluate the impact of a SCAMP for chest pain on practice variation and resource utilization.

**Methods and Results:**

We compared demographic variables, clinical characteristics, and cardiac testing in a historical cohort (n=406) of patients presenting to our outpatient division for initial evaluation of chest pain in the most recent pre-SCAMP calendar year (2009) to patients enrolled in the chest pain SCAMP (n=364). Demographic variables including age at presentation, sex, and clinical characteristics were similar between groups. Adherence to the SCAMP algorithm for echocardiography was 84%. Practice variation decreased significantly after implementation of the SCAMP (*P*<0.001). The number of exercise stress tests obtained was significantly lower in the SCAM*P*-enrolled patients compared with the historic cohort (∼3% of patients versus 29%, respectively; *P*<0.001). Similarly, there was a 66% decrease in utilization of Holter monitors and 75% decrease in the use of long-term event monitors after implementation of the chest pain SCAMP (*P*=0.003 and *P*<0.001, respectively). The number of echocardiograms obtained was similar between groups.

**Conclusions:**

Implementation of a SCAMP for evaluation of pediatric chest pain has lead to a decrease in practice variation and resource utilization. **(*J Am Heart Assoc*. 2012;1:jah3-e000349 doi: 10.1161/JAHA.111.000349.)**

## Introduction

The outpatient management of chest pain in children is often resource intensive and costly despite the exceedingly low incidence of cardiac pathology.^1–9^ Widespread practice variation among pediatric cardiologists contributes to this phenomenon in part because of the lack of evidence-based standards for the evaluation of pediatric chest pain. Therefore, we created a standardized approach to pediatric chest pain that has been implemented across our department as part of a broader quality improvement initiative, which we have termed Standardized Clinical Assessment and Management Plans (SCAMPs).

SCAMPs is a novel quality improvement methodology that standardizes the assessment and management of a relatively diverse patient population with a single presenting symptom or condition and incorporates a systematic yet selective data collection process.^10^ On the basis of periodic review of the collected data, the SCAMP algorithm is designed to be modified. The goals of the SCAMPs initiative, including the chest pain SCAMP, are to improve patient care, decrease practice variation, and reduce unnecessary resource utilization and cost.

Our previous analyses have predicted that utilization of several diagnostic tests including echocardiograms, exercise stress tests (ESTs), and outpatient rhythm monitors could be substantially reduced using the chest pain SCAMP leading to an ∼20% reduction in costs without negatively affecting patient care.^11^ Our objective of this study was to evaluate the effect of the chest pain SCAMP on practice variation and resource utilization.

## Methods

As detailed in prior work from this institution, we developed an algorithm that forms the basis of the chest pain SCAMP using history, physical examination, and ECG to suggest when further diagnostic testing including an echocardiogram, EST, or outpatient rhythm monitor is indicated.^11^ This algorithm is targeted at identifying cardiac causes of chest pain. Testing recommendations were based on a recent report from Kane et al.^6^ Over a 10-year period, there were 32 cases of serious underlying cardiac pathology presenting with chest pain to our outpatient cardiology department. By retrospective review, all of these cardiac diagnoses could have been identified by using history, physical examination, ECG, or echocardiography alone, without the use of other diagnostic tests such as EST or outpatient rhythm monitors.

### Patient Selection

All patients between 7 and 21 years of age presenting to our outpatient pediatric cardiology clinic for a first-time evaluation of chest pain were enrolled in the chest pain SCAMP. Patients enrolled in the SCAMP from June 1, 2010, through May 31, 2011, were included in this analysis. Children with a known history of heart disease were excluded. We previously reported on a similar cohort of patients (historical cohort) who presented for initial evaluation of chest pain in 2009 (the most recent pre-SCAMP year), identified by International Classification of Diseases, Ninth Revision (ICD-9) billing codes for chest pain.^11^ Patients in the historic cohort were similarly excluded if they had a known history of heart disease or if they had a prior evaluation for chest pain by a pediatric cardiologist. The Institutional Review Board for Clinical Research at Children's Hospital Boston approved the use of patient medical records for the retrospective review.

### Clinical Characteristics

We collected demographic and clinical characteristics for each patient including historical features of the chest pain and associated symptoms, past medical history, family history, physical examination findings, and electrocardiographic results. For SCAMP-enrolled patients, these data were collected on SCAMP data forms completed by the provider at the time of the visit. For patients in the historic cohort, the same data were retrospectively ascertained from the cardiologist's clinic note created at the time of the visit.

To target potential cardiac causes of chest pain, pertinent positive clinical history included whether the pain was associated with exertion or exertional syncope, radiated to the back, jaw, left arm, or left shoulder, increased with supine position, or was temporally associated with fever. Past medical history was considered positive if the patient had a condition that could lead to an increased risk of pathologic chest pain including systemic arthritis/vasculitis, a hypercoaguable state, or prolonged immobilization. Family history was considered positive if any of the following were present in a first-degree relative: sudden or unexplained death, cardiomyopathy, or a hypercoaguable state. Pertinent positives on physical examination included a pathological murmur, gallop, pericardial friction rub, abnormal second heart sound, distant heart sounds, peripheral edema, painful or swollen extremities, tachypnea, or fever (oral temperature >38.4°C).

### Test Interpretation

Electrocardigraphic interpretation was based on documented findings in the cardiologist's clinic note in the historical cohort and on the SCAMP data form for the SCAMP cohort. Ventricular hypertrophy, pathological ST-segment or T-wave changes (>2 mm), high-grade atrioventricular block, ventricular or atrial ectopy, low QRS voltages, PR segment depression, S1/Q3/inverted T3, or a prolonged QTc>470 ms were considered positive criteria.

Echocardiography, EST, and Holter and long-term event monitor results were obtained from reports generated at the time of the study in the historical cohort and from the SCAMP data form in the SCAMP group. Testing was generally obtained at the time of or soon after the initial visit for chest pain. Diagnoses from these tests that were considered potential cardiac causes of chest pain included specific coronary anomalies, cardiomyopathy, myocarditis, pericarditis, pulmonary hypertension, aortic dissection, and pulmonary embolism.

### Adherence, Practice Variation, and Resource Utilization

We analyzed adherence to the SCAMP algorithm in terms of echocardiography. We examined how often providers ordered an echocardiogram when indicated by the SCAMP as well as how often they followed recommendations not to order an echocardiogram when it was not indicated by the SCAMP. Practice variation for diagnostic testing was also determined (see statistical analysis). Variation was assessed at the patient level. Patients were seen by 34 providers in the historic cohort (median number of patients per physician 5.5, 1–74) and 36 providers in the SCAMP cohort (median number of patients per physician 4, range 1 to 78) during the study period. The number of cardiac tests obtained in conjunction with the clinic visit was analyzed. All patients in both cohorts had at least 1 cardiology clinic visit. An ECG was performed at all clinic visits. Resource utilization for echocardiograms, ESTs, Holter monitors, and event monitors was compared between the 2 groups.

### SCAMP Deviation Analysis

Explanations for deviations from the SCAMP were collected from data provided at the time of the visit either in the SCAMP data form or in the clinic note. For echocardiograms that were not recommended by the SCAMP but were still ordered, reasons for deviation were categorized into the following: parental concern, underlying medical illness (distinct from those outlined in past medical history previously), abnormal physical examination finding (unlikely to be related to chest pain), or other.

### Statistical Analysis

Demographic and clinical characteristics were compared between groups using Fisher's exact or the Wilcoxon rank sum test as appropriate. Resource utilization was compared between groups using Fisher's exact test.

**Figure 1.**
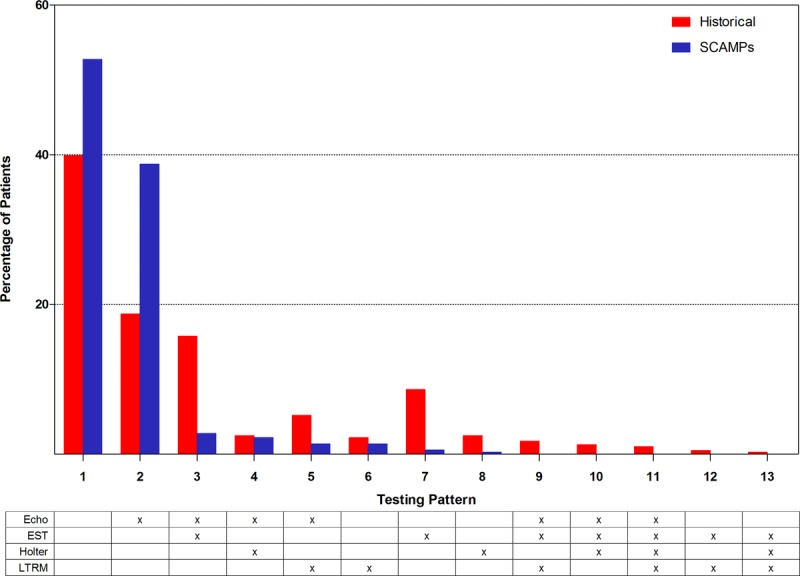
Practice variation in diagnostic testing before and after chest pain SCAMP (*P*<0.001). SCAMP indicates Standardized Clinical Assessment and Management Plans; Echo, echocardiogram; EST, exercise stress test; LTRM, long-term rhythm monitor.

We assessed practice variation in the use of echocardiography, EST, Holter, and event monitoring at the encounter level between the 2 time periods as follows. There were 16 possible testing combinations using these 4 tests (Figure 1). In the case of random choice of testing, one would expect to observe all 16 combinations. However, as the variation in the choice of testing decreases, the number of observed testing combinations would also decrease. The difference in the number of combinations observed in the SCAMP and historical cohorts served as a measure of change in practice variation. Bootstrapping was used to calculate the *P* value for this test statistic.

Explanations for SCAMP deviations were expressed as counts and percentages. All statistical analysis were 2-sided and type I error was controlled at a level of 0.05. Analyses were performed with SPSS (version 19.0, SPSS Inc, Chicago, IL) and S-Plus (Version 8.0, TIBCO Software, Palo Alto, CA).

## Results

Demographic variables were similar between the historical cohort and the SCAM*P*-enrolled patients (Table 1). In both groups, boys and girls were evenly split, the majority between 12 and 16 years of age. The groups were also similar with respect to presenting data such as exertional chest pain, relevant past medical history, and family history. Patients in the SCAMP cohort had a higher incidence of associated palpitations. There were no differences between groups in terms of abnormal physical examination findings or pertinent abnormalities on ECG.

**Table 1:** Demographic and Clinical Data

	Historical Cohort (n=406)	SCAMP Cohort (n=364)	*P* value
Male (n, %)	207 (51)	187 (51)	0.94

Age (median, range in y)	13.7 (7–21)	13 (7–19)	0.07

Age 7–11, y (%)	118 (29)	137 (38)	<0.0001

Age 12–16, y (%)	184 (45)	179 (49)	

Age 17–21, y (%)	104 (26)	48 (13)	

Exertional chest pain	151 (37%)	141 (39%)	0.71

Associated palpitations	66 (16%)	90 (25%)	0.004

Positive past medical history*	2 (0.5%)	5 (1%)	0.27

Positive family history†	4 (1%)	9 (2%)	0.16

Abnormal physical examination‡	6 (1%)	3 (1%)	0.51

Abnormal ECG¶	12 (3%)	5 (1%)	0.15

SCAMP indicates Standardized Clinical Assessment and Management Plans.

* Positive past medical history indicates: systemic arthritis/vasculitis, hypercoaguable state, or prolonged immobilization.

† Positive family history indicates: sudden or unexplained death, cardiomyopathy, or a hypercoaguable state in first-degree relative.

‡ Abnormal physical examination indicates: pathological murmur, gallop, pericardial friction rub, abnormal second heart sound, distant heart sounds, peripheral edema, painful or swollen extremities, tachypnea, or fever.

¶ Abnormal ECG indicates: ventricular hypertrophy, pathological ST-segment or T-wave changes (>2 mm), high-grade atrioventricular block, ventricular or atrial ectopy, low QRS voltages, PR segment depression, S1/Q3/inverted T3, or prolonged QTc>470 ms.

Overall, the majority of providers followed the SCAMP algorithm with regards to the use of echocardiography. Specifically, in clinical encounters where an echocardiogram was recommended on the basis of the SCAMP, this recommendation was adhered to in 84.4% (95% CI, 78.7–90.1) of cases. When an echocardiogram was not indicated by the SCAMP, providers followed the recommendation not to obtain an echocardiogram 83.8% (95% CI, 78.8–88.8) of the time.

Analysis of practice variation showed that 13 of the possible 16 testing patterns were observed in the historical cohort. The largest proportion of patients (40%) underwent no testing in addition to ECG, while the majority of the remaining patients underwent echocardiogram only (19%), EST only (9%), or the combination of the two (16%). In the SCAMPs cohort, only 8 testing patterns were observed; the majority of patients underwent no additional testing (53%) or echocardiogram only (39%). This represented a statistically significant reduction in practice variation in the SCAMP cohort compared with the historic cohort (*P*<0.001) (Figure 1).

**Figure 2.**
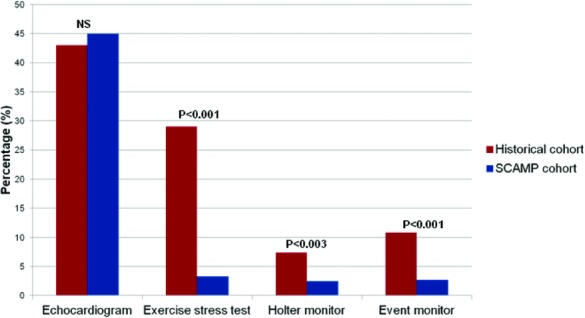
Resource utilization by diagnostic test before and after chest pain SCAMP. SCAMP indicates Standardized Clinical Assessment and Management Plans.

The decrease in practice variation with regard to diagnostic testing translated into a reduction in resource utilization for the majority of testing modalities. Exercise stress testing was markedly lower in the SCAMP cohort than in the historical cohort (3.3% versus 29%, *P*=0.001). Holter monitor (2.5% versus 7.3%, *P*=0.002) and long-term event monitor (2.7% versus 10.8%, *P*=0.001) utilization was also lower in the SCAMP cohort. There was no difference in echocardiography utilization between the 2 groups (Figure 2).

Although there was no difference in overall echocardiogram utilization, there was a trend toward more appropriate utilization. In patients with exertional chest pain (where an echocardiogram is generally indicated), the number of patients who did not have an echocardiogram was lower in the SCAMP group compared with the historic cohort (∼14% versus 38%, *P*<0.0001) (Table 2). Additionally, of those patients with chest pain predominantly at rest and without other concerning features of their past medical history, family history, physical examination, or ECG (ie, patients who generally would not warrant echocardiographic investigation), 28% in the historic cohort had an echocardiogram compared with only 15% in the SCAMP cohort (*P*=0.001) (Table 2).

**Table 2.** Appropriateness of Echocardiographic Utilization

	Historical Cohort (% {95% CI})	SCAMP Cohort (% {95% CI})	*P* value
Patients with exertional chest pain who did not have an echocardiogram	58/151	19/141	<0.0001

	(38.4 {30.7–46.2})	(13.5 {7.8–19.1})	

Patients with chest pain only at rest without other concerning features who had an echocardiogram	71/255	34/233	0.001

	(27.8 {22.3–33.3})	(15.2 {10.5–20.0})	

SCAMP indicates Standardized Clinical Assessment and Management Plans.

Provider explanations for deviations from the chest pain SCAMP with regard to echocardiograms are shown in Table 3. Parental concern, other coexisting medical conditions (not necessarily associated with causing cardiac chest pain in children), and abnormal physical examination findings unrelated to cardiac chest pain occurred equally as the dominant reasons for deviation.

**Table 3.** Echocardiographic Deviations (Echocardiograms Ordered Despite SCAMP Recommendations)

Reason for Deviation	No. (%)
Parental concern	4 (31)

Underlying medical illness (other than those listed in SCAMP past medical history)*	4 (31)

Abnormal physical examination finding (not necessary related to chest pain)†	4 (31)
Other (echocardiogram ordered by primary medical physician prior to evaluation)	1 (8)

SCAMP indicates Standardized Clinical Assessment and Management Plans.

* History of acute lymphoblastic leukemia (ALL), connective tissue disorder (2), and eating disorder.

† Ejection click (2), murmur suggestive of atrial septal defect, fixed split S2.

Importantly, no abnormalities were detected on cardiac evaluation that represented a cardiac etiology for chest pain. There were, however, several incidental diagnoses discovered by echocardiography including 2 patients with a bicuspid aortic valve and 1 patient with a large secundum atrial septal defect. Two children were also found to have a high origin of the right coronary artery near the sinotubular junction. The take-off in 1 of these patients was from a somewhat leftward aspect of the right sinus of Valsalva with an origin that was somewhat acutely angled, although considered unlikely to be the cause of this child's single episode of chest pain.

## Discussion

Chest pain is a common problem in pediatrics and a frequent reason for referral to a pediatric cardiologist. In contrast to adult populations, chest pain in childhood is rarely related to cardiac pathology. Despite this distinction, the diagnostic approach to pediatric chest pain varies considerably among clinicians without any clear consensus. In this study, we demonstrate a reduction in practice variation and resource utilization using a SCAMP for the evaluation of pediatric chest pain.

Practice variation contributes to inefficiency and cost in healthcare delivery. This variation has been well documented in the management of a broad range of disease processes over the past few decades. In Medicare patients, for instance, adjusted per capita spending in the year 2000 was more than double in Manhattan, NY, than in Portland, OR. Importantly, however, high-intensity practice patterns amongst Medicare patients (in part characterized by the number of diagnostic tests performed) were associated with lower quality care and worse adjusted outcomes than more conservative practice patterns.^12^ SCAMPs represent a potential unique algorithm-based solution in which understanding and controlling variation can lead to consistent behavior where outcomes can be measured and practice can be systematically improved based on analysis of sound deviations.

SCAMPs were developed as part of a broader quality improvement initiative to reduce practice variation in clinical decision making, but unlike clinical practice guidelines or other protocol-driven algorithms, SCAMPs have several unique features that distinguish them from these previously established methodologies. The inception of SCAMPs is in part related to a lack of evidence-based guidelines and difficulty in conducting large-scale, prospective, randomized clinical trials within our specialty which often form the basis of algorithms such as clinical practice guidelines. Unlike other quality improvement tools, SCAMPs are applied to conditions where there is no “best” practice but only “sound” practice based on existing data in combination with expert clinical consensus. The SCAMPs process is iterative with rigorous data collection and timely revision of guidelines based on these data. Accordingly, SCAMPs are primarily focused on the content of guidelines as opposed to the process of executing evidence-based guidelines. The SCAMPs process allows for and in fact encourages deviations in care plan based on knowledge-based preferences with the only requirement that deviations are explained in order to improve the care algorithm.^10^ Pediatric chest pain is particularly suited for this type of analysis given the wide variation in clinical decision making, lack of evidence-based guidelines based on rigorous analytic study, the low incidence of true cardiac pathology, as well as the extensive resource utilization and cost related to its management.

In this study, we focused on a SCAMP applied to pediatric patients presenting specifically to our outpatient cardiology clinic with chest pain. Several studies have attempted to characterize this patient population, though to our knowledge this is the first study to implement a SCAMP-type process. In general, other authors have also found that echocardiograms and other diagnostic tests are obtained in a significant number of children presenting with chest pain with low yield in identifying a cardiac etiology.^1–9^ Incidental diagnoses, however, are not uncommonly found by diagnostic cardiac tests such as echocardiography or outpatient rhythm monitoring.^1,3,5,9^

Provider adherence to the chest pain SCAMP for echocardiography was approximately 84% and this contributed to a significant decrease in practice variation. As noted above, given the iterative nature of the SCAMP algorithm, perfect adherence is not the goal. Resource utilization significantly decreased for a number of diagnostic testing modalities commonly used in the evaluation of chest pain. Most notably, in the year prior to the implementation of the chest pain SCAMP, nearly 30% of patients presenting to our outpatient cardiology department with chest pain underwent exercise stress testing despite a lack of established clinical utility demonstrated in several studies.^2,8,9,13^ In the SCAMP period, utilization of this test decreased to just over 3% of patients. Significant reductions were also seen in the utilization of outpatient rhythm monitoring devices including Holter and event monitors.

Translating reductions in resource utilization into actual cost savings is challenging and requires several assumptions. However, using a blended cost/charge ratio of 60% and assuming that an equivalent number of patients had presented to our clinic after SCAMP implementation, our reductions in diagnostic testing could have lead to potential cost savings of over $160 000 during this time period. As national concerns surrounding healthcare financing continue, novel methods of decreasing healthcare spending while delivering high-quality patient care will be increasingly essential.

Despite the SCAMP algorithm, the utilization of echocardiography did not change significantly though there appeared to be a trend toward more appropriate utilization of echocardiography with regards to its use in exertional and nonexertional chest pain. In our prior analysis, when the chest pain SCAMP was theoretically applied to the historical cohort of patients, we predicted an approximately 20% decrease in echocardiogram utilization.^11^ A number of factors may account for the continued higher use of echocardiography. First, the vast majority of echocardiograms were obtained based on historical features of the chest pain, particularly if the pain was exertional in nature. The initial chest pain SCAMP did not specifically define “exertional chest pain” and thus resulted in variable interpretation of this characteristic.

Additionally, providers cited a variety of reasons for ordering echocardiograms not indicated by the SCAMP. For instance, several providers obtained echocardiograms to alleviate parental anxiety. In several other patients, coexisting medical conditions (eg, leukemia, concern for connective tissue disease, anorexia) or physical examination findings felt to be unrelated to chest pain (eg, a click or a fixed split second heart sound suggestive of an atrial septal defect) were noted as reasons for obtaining an echocardiogram. Even with substantial reduction of some studies such as exercise stress testing and outpatient arrhythmia monitoring, echocardiography remains a heavily used resource with no yield of a cardiac etiology for chest pain during the SCAMP study period. Further modifications of the SCAMP may potentially lead to a reduction of echocardiogram utilization in the future.

In addition to the financial benefits achieved in reducing unnecessary testing, this SCAMP may have a positive psychosocial effect by decreasing patient and parental anxiety that is generated in part by additional testing.^14^ This can occur while awaiting test results or as a result of identifying anatomic variants or anomalies unrelated to chest pain that have no known clinical impact (eg, high right coronary from sinotubular junction). Despite reassurance and appropriate counseling, these diagnoses can be anxiety provoking and lead to potential behavior modification of a child's activities or participation in athletics. Additionally, some families may have been reassured by the fact that the provider was following a set of expert consensus guidelines used by our entire department. In this regard, the SCAMP may facilitate a discussion as to why a particular patient may not warrant additional testing.

Despite our findings, this study has several limitations. Data collection was accomplished using retrospective review in the historical cohort whereas we used a form completed by the physician at the time of encounter in SCAMP patients. This difference may account for the increased frequency of associated complaints such as palpitations reported in the SCAMP cohort and may overestimate our perceived improvement in more appropriate echocardiography usage. Second, although we only identified a few cardiac causes of chest pain in our historical group (pericarditis), no cardiac etiology for chest pain was identified in the current SCAMP group. It is possible that we have missed pathology, which may present in alternative settings such as an emergency department or with longer-term follow-up. Notably however, a recent report from our institution found that of 3700 pediatric patients presenting with chest pain, there were no deaths attributable to cardiac pathology over a median follow-up of 4.4 years.^1^ Moreover, as noted previously, the 37 patients with a potential cardiac etiology for chest pain would have all been identified using the chest pain SCAMP.^6^ Furthermore, we cannot account for additional resource utilization for patients seeking further evaluation after an initial visit in our outpatient clinic, an area that will be the focus of future investigation. The ability to generalize the effect of the chest pain SCAMP to other settings may be limited as our population is prescreened and referred for a higher suspicion of a cardiac etiology for chest pain. Lastly, patients with significant cardiac pathology may be incompletely captured if the referred chief complaint is different from chest pain and chest pain is elicited secondarily. Despite these limitations, this preliminary study demonstrates that the SCAMPs process can streamline provider practice patterns and reduce variation in diagnostic testing.

## Conclusions

In this study, we report a significant decrease in practice variation and resource utilization after implementation of a novel quality improvement methodology for pediatric chest pain. The use of echocardiography remains largely unchanged despite predicted decrease. Future iterations of the chest pain SCAMP can potentially refine data collection and recommendations to reduce echocardiogram use without increasing missed diagnoses.
